# The impact of three amino acids with distinct electrical properties on the synthesis of α-casein by MAC-T cells

**DOI:** 10.1038/s41598-025-29015-0

**Published:** 2025-12-29

**Authors:** Yu Ding, Lewen Xie, Min Yang, Xinyu Zhang, Liang Yang, Wanping Ren, Ruoshan Luo, Yang Yang, Hang Zhang, Wei Shao

**Affiliations:** College of Animal Science, Xinjiang Agricultural University, Urumqi, 830052 China

**Keywords:** Proline, Glutamic acid, Serine, MAC-T cells, α-Casein, Cell biology, Molecular biology

## Abstract

This study utilized in vitro cultured bovine mammary epithelial cells line(MAC-T cells) to examine how different concentrations of non-polar proline (Pro), polar negatively charged glutamic acid (Glu), and polar uncharged serine (Ser) affect α-casein synthesis. A multi-factor level completely randomized experimental design was adopted. MAC-T cells were cultured in vitro using a medium containing 10% fetal bovine serum (10% FBS DMEM). After adhering to the wall, the cells were starved in serum-free medium for 12 h. The cells in the serum-free medium group were used as the blank control group (CK, 0× group), and the experimental groups were respectively supplemented with Pro (0.52, 1.04, 2.09, 4.17, 8.35, 16.69 mmol/L), Glu (0.77, 1.53, 3.06, 6.13, 12.25, 24.50 mmol/L), and Ser (0.50, 0.99, 1.97, 3.93, 7.86, 15.71 mmol/L). The proliferation rate of MAC-T cells was detected by CCK8 method; the synthesis amount of α-casein inside and outside MAC-T cells was detected by enzyme-linked immunosorbent assay; the expression levels of genes related to the synthesis of α-casein in MAC-T cells were detected by real-time fluorescence quantitative PCR (RT-qPCR) technology. The addition of Glu had the highest proliferation rate and the most significant effect at 12 h. The addition of 16.69 mmol/L Pro, 24.50 mmol/L Glu, and 0.99 mmol/L Ser could significantly increase the synthesis of α-casein (*P* < 0.01). When Pro, Glu, and Ser were added respectively, they all promoted the up-regulation of the expression of α-casein synthesis-related genes CSN1S1 and CSN1S2 in MAC-Tcells. Amino acids with distinct charge natures (acidic Glu, polar Ser, non-polar Pro) all exhibited positive regulatory roles in the synthesis of α-casein in MAC-T cells. Among them, the non-polar amino acid proline had the most pronounced promoting effect, disclosing that the disparity in charge characteristics is a crucial regulatory factor affecting the synthesis of α-casein.

**Supplementary Information:**

The online version contains supplementary material available at 10.1038/s41598-025-29015-0.

## Introduction

[Research Significance] Casein (αs1CN, αs2CN, βCN, κCN) accounts for over 80% of the protein in milk^1^. Over 90% of the protein in cow’s milk is de novo synthesized by mammary epithelial cells, with the raw materials mainly being free amino acids from the blood^2^. Amino acids are not only the key substrates for protein synthesis but also act as signal molecules that regulate casein synthesis, making their composition and ratio particularly important^3,4^. Moreover, the influence of amino acids on the synthesis of MAC-T cells α-casein is a multi-faceted biological process, which not only relates to the basic units of protein synthesis but also involves multiple aspects such as cell signal transduction and metabolic regulation^5,6^. The electrical property of amino acids refers to the charge state of the amino acid molecule under specific pH conditions, mainly determined by their side chains (R groups). The side chain electrical properties of amino acids—including non-polar, polar uncharged, and charged (positively or negatively) groups—can differentially influence the metabolic activities of mammary epithelial cells through various mechanisms, such as modulating the pH, ion homeostasis, or transmembrane transport efficiency within the cellular microenvironment. These properties significantly affect protein solubility, stability, charge distribution, and molecular interactions^7–10^. However, it remains unclear whether, and by what mechanisms, specific amino acids with distinct intrinsic electrical characteristics differentially regulate the proliferation and milk protein synthesis in MAC-T cells. Mammary epithelial cells transport free amino acids from the blood into the cells through specific transport mechanisms to participate in the synthesis of milk proteins and casein^11^. Therefore, in-depth exploration of the electrical properties and ratio patterns of amino acids in dairy cows and their effects on the synthesis of casein by mammary epithelial cells is of great significance. [Previous Research Progress] Amino acids not only directly participate in the synthesis of milk proteins as substrates^12^, but also function as key cellular signaling molecules, playing critical regulatory roles in signal transduction by modulating cell proliferation, activating major signaling pathways, and regulating the expression of amino acid transporters^13–18^. Studies have shown that positively charged amino acids (such as arginine) may promote protein synthesis by activating the mTOR signaling pathway^19^. On the other hand, negatively charged amino acids (such as glutamic acid) may affect protein folding and stability by regulating intracellular pH^20^. The unique cyclic structure of the nonpolar amino acid proline confers substantial conformational rigidity, which not only influences protein secondary structure but also, as recent studies indicate, may play a specialized role in cellular proliferation and stress response pathways^21^. Glutamic acid, a negatively charged residue at physiological pH, is critical for mediating ionic interactions, regulating intracellular pH homeostasis, and serving as a precursor for essential metabolites—functions that can directly influence cellular anabolism^22^. Serine, a polar uncharged amino acid containing a reactive hydroxyl group, is highly susceptible to post-translational modifications such as phosphorylation and serves as a key contributor to the biosynthesis of phospholipids and one-carbon units, processes that are vital for the function and integrity of mammary epithelial cells^23^. [Research Entry Point] In this study, positively charged proline (Pro), negatively charged glutamic acid (Glu), and uncharged serine (Ser) were supplemented into the culture of MAC-T cells. Cell proliferation, α-casein synthesis levels, and the expression of α-casein synthesis-related genes, including αs1-casein (CSN1S1) and αs2-casein (CSN1S2), were assessed using the MTT cytotoxicity assay, enzyme-linked immunosorbent assay (ELISA), and real-time quantitative PCR (RT-qPCR), respectively. [Problems to be Solved] To verify whether the addition of different electrically charged amino acids at single levels would affect the proliferation of mammary epithelial cells and the synthesis of α-casein.

## Results

### Effect of adding three amino acids with different electrical properties on the proliferation rate of MAC-T cells

**Fig. 1:**
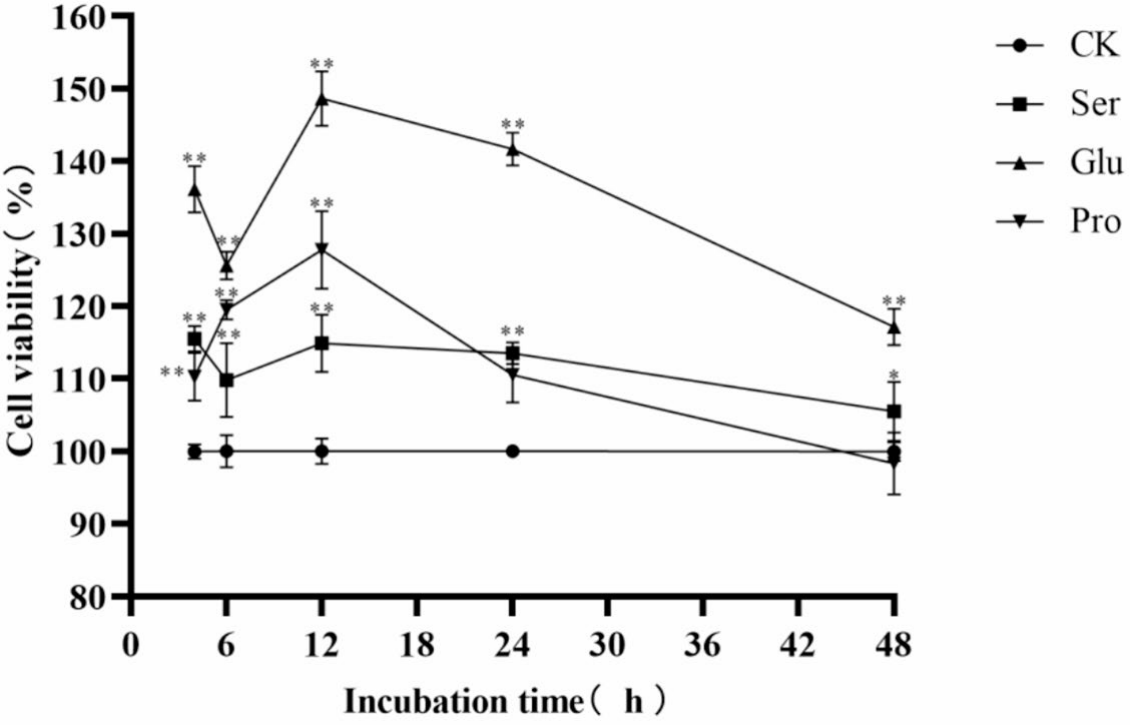
Effects of Different Levels of Pro, Glu and Ser on the Proliferation Rate of MAC-T Cells. The error bars represent the SD (*n* = 3). ANOVA was used to detect the differences between groups, and the LSD method was combined for uncorrected exploratory pairwise comparisons, while the Duncan method was used for conservative multiple comparison corrections. The CK group represents the blank control group; in the figure, * indicates *P* < 0.05, and ** indicates *P* < 0.01.

As shown in Fig. 1, compared with the CK group, the addition of Pro, Glu, and Ser alone at 4 h, 6 h, 12 h, and 24 h had extremely significant effects on the proliferation rate of MAC-T cells (*P* < 0.01); at 48 h, only Glu had an extremely significant effect o n the proliferation rate of MAC-T cells (*P* < 0.01), while Ser had a significant effect (*P* < 0.05) and Pro had no significant effect. When compared among the groups, there was a highly significant difference (*P* < 0.01) in the proliferation rate of the negatively charged polar Glu compared with the non-polar Pro and the polar uncharged Ser, its proliferation effect was the best. The proliferation rate of each experimental group reached the maximum at 12 h, to guarantee that the cell state tended to be stable after the addition of amino acids, samples were collected at 12 h when different levels of Pro, Glu, and Ser were added.

### Effect of adding three amino acids with different electrical properties on the amount of α-casein synthesized

**Fig. 2:**
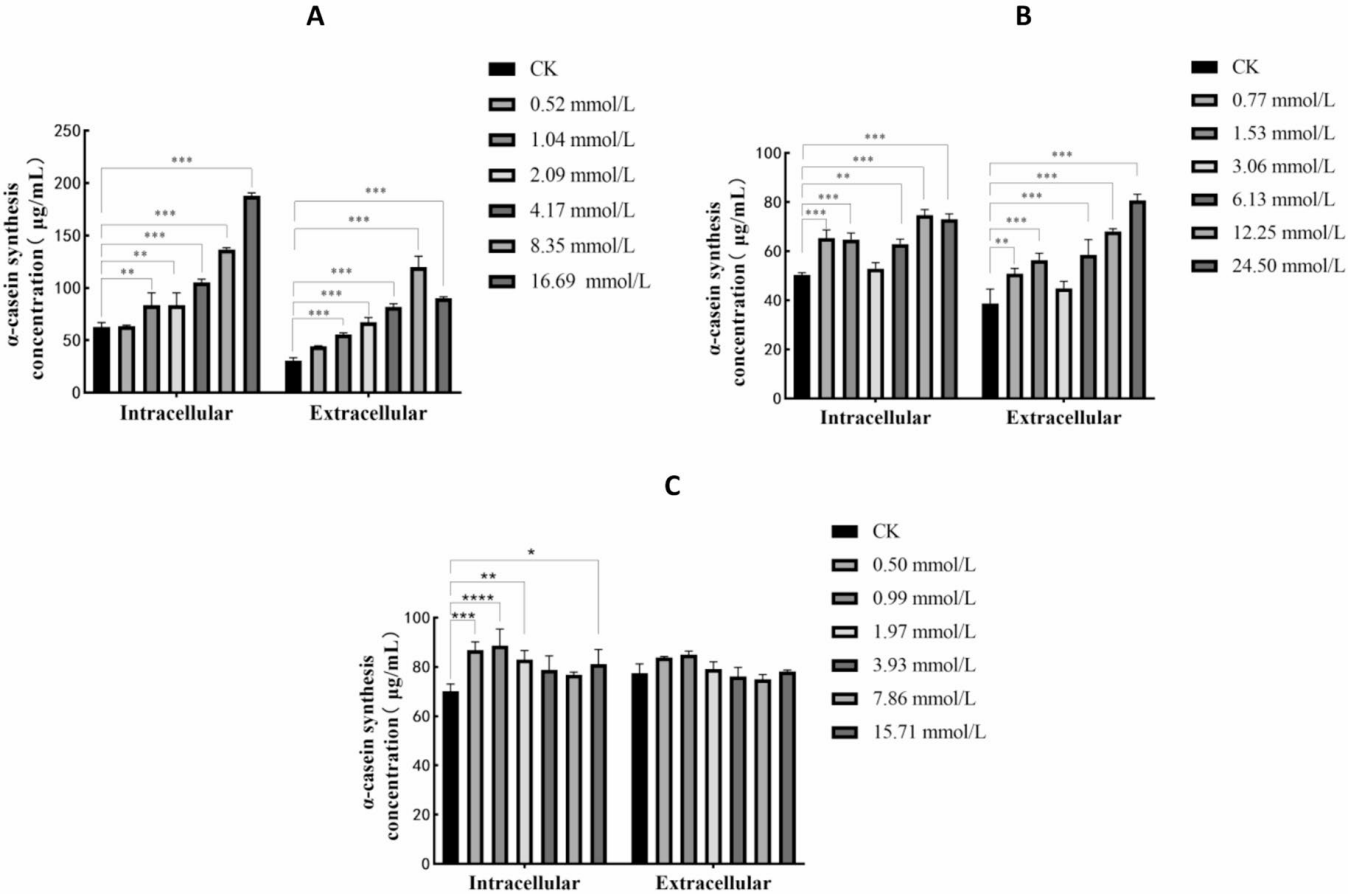
Effect of single addition of different levels of Pro, Glu and Ser on the synthesis of intracellular and extracellular α-casein. (**A**–**C**) represent the effect of different concentrations of Pro, Glu and Ser on the amount of intra- and extracellular α-casein synthesis. The error bars represent the SD (*n* = 3). ANOVA was used to detect the differences between groups, and the LSD method was combined for uncorrected exploratory pairwise comparisons, while the Duncan method was used for conservative multiple comparison corrections. The CK group represents the blank control group; in the figure, * indicates *P* < 0.05, ** indicates *P* < 0.01, and *** indicates *P* < 0.001, **** indicates *P* < 0.0001.

The culture medium and cells were collected at 12 h after adding Pro, Glu and Ser with different electrical properties and different levels. As shown in Fig. 2, the addition of Pro, Glu and Ser alone had significant effects on the synthesis of α-casein in cells. When non-polar Pro was added, except for the 0.25× group (0.52 mmol/L Pro), which had no significant effect on the synthesis of α-casein in the intracellular fluid and cell supernatant (*P* > 0.05), the other groups all had significant effects on the synthesis of α-casein in the intracellular fluid and cell supernatant (*P* < 0.01). When polar and negatively charged Glu was added, except for the 1× group (3.06 mmol/L Glu), which had no significant effect on the synthesis of α-casein in the intracellular fluid and cell supernatant (*P* > 0.05), the other groups all had extremely significant effects on the synthesis of α-casein in the intracellular fluid and cell supernatant (*P* < 0.01). When polar uncharged Ser was added, no significant effect was observed on the synthesis amount of extracellular α-casein (*P* > 0.05). The 2× group (3.93 mmol/L Ser) and the 4× group (7.86 mmol/L Ser) had no significant influence on the synthesis amount of intracellular α-casein (*P* > 0.05), the 8× group (15.71 mmol/L Ser) significantly affected the synthesis amount of intracellular α-casein (*P* < 0.05), the remaining groups all had extremely significant effects on the synthesis amount of intracellular α-casein (*P* < 0.01).

### Effect of adding three amino acids with different electrical properties on the expression of genes related to α-casein synthesis

**Fig. 3:**
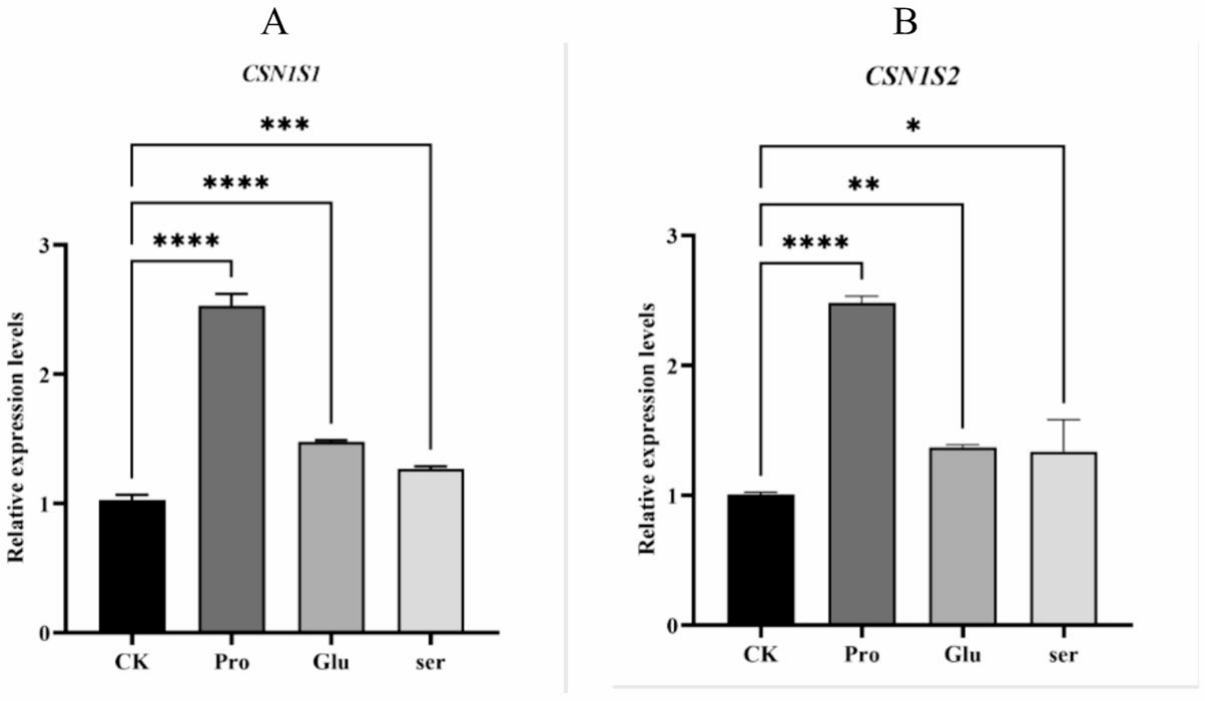
Effect of single addition of Pro, Glu and Ser on the expression of genes related to α-casein synthesis. (**A**,**B**) represent the relative mRNA expression levels of CSN1S1 and CSN1S2, respectively. The error bars represent the SD (*n* = 3). ANOVA was used to detect the differences between groups, and the LSD method was combined for uncorrected exploratory pairwise comparisons, while the Duncan method was used for conservative multiple comparison corrections. The CK group represents the blank control group; in the figure, * indicates *P* < 0.05, ** indicates *P* < 0.01, and *** indicates *P* < 0.001, **** indicates *P* < 0.0001.

As shown in Fig. 3, the addition of different charged amino acids, Pro, Glu, and Ser, each had a highly significant effect on the expression level of the α-casein synthesis-related gene CSN1S1 (*P* < 0.001). The addition of non-polar Pro and negatively charged Glu had a highly significant effect on the expression level of CSN1S2 (*P* < 0.01), while the addition of uncharged Ser had a significant effect on the expression level of CSN1S2 (*P* < 0.05). Among them, the relative expression levels of CSN1S1 and CSN1S2 in the Pro addition group were highly significantly higher than those in the Glu and Ser addition groups (*P* < 0.01).

## Discussion

### Effect of adding three amino acids with different electrical properties on the proliferation rate of MAC-T cells

The growth and proliferation of cells in animals necessitate various nutrients, including amino acids, vitamins, hormones, and growth factors^24,25^. Cell proliferation requires the synthesis of a substantial quantity of new proteins to support the development of cellular structure and function^26^. As fundamental building blocks for protein synthesis, amino acids are indispensable for sustaining rapid cell division and growth. An optimal temporal and concentration-dependent relationship exists for amino acids in promoting cell proliferation; deviations from this ideal range, either below or above, lead to reduced efficacy^27–29^. This study demonstrates that the stimulatory effects of proline (Pro), glutamic acid (Glu), and serine (Ser) on cell proliferation are maximized when these amino acids are present at specific concentrations and maintained for an optimal duration of 12 h.

The electrical nature of amino acids may exert an influence on cell proliferation by affecting cellular metabolism and signal transduction pathways. Electrical stimulation, as an external factor, can modulate the proliferative behavior of cells, while the reprogramming of amino acid metabolism may also indirectly impact cell proliferation through altering the intracellular environment^30^. The results of this experiment demonstrate that the proliferation effect of polar and negatively charged acidic glutamic acid is the most remarkable. Especially at high concentrations, it can significantly enhance the cell proliferation. This might be attributed to the negatively charged side chain group in its structure, which is an acidic amino acid. Research indicates that cancer cells are sensitive to acidic microenvironments, and such microenvironments can facilitate the survival and invasion of cancer cells^31^. During the process of cell proliferation, oxidative stress may arise. Acidic amino acids, serving as antioxidants or constituents of the antioxidant defense system, might contribute to alleviating the damage of oxidative stress to cells, thereby facilitating the cell proliferation process. Negatively charged amino acids could function as receptors or ligands of signaling molecules, participating in the intracellular signal transduction processes and directly or indirectly influencing the proliferation rate of cells. Secondly, polar uncharged serine significantly enhanced the proliferation of MAC-T cells as well. Serine is a vital precursor of phospholipids such as phosphatidylserine (PS), and phospholipids are the major constituents of cell membranes^32^. The stability of the cell membrane is of paramount importance for cell survival. Serine might indirectly modulate the cell’s responses to external signals by influencing the fluidity of the cell membrane and the distribution of receptors, thereby affecting the cell proliferation rate. Meanwhile, non-polar proline exerts a significant promoting effect on the proliferation of MAC-T cells. Non-polar amino acids typically remain uncharged at physiological pH and are hydrophobic, being crucial for the stability of protein structure. Consequently, they might indirectly influence cell proliferation through regulating physiological functions, metabolic actions, and providing energy.

Research has demonstrated that the negatively charged amino acid glutamic acid exhibits potential in promoting the proliferation of MAC-T cells. It is hypothesized that during the perinatal period in dairy cows, when mammary tissue requires repair and regeneration due to infection (e.g., mastitis), dietary supplementation with glutamic acid may support the renewal of mammary epithelial cells. This could contribute to improved mammary health and enhanced production performance in subsequent lactation periods. Currently, there is limited research on the direct impact of amino acid electrical properties on cell proliferation rates. It is hypothesized that these electrical properties may influence interactions between amino acids and cellular components in the membrane, cytoplasm, or nucleus, potentially modulating intracellular metabolic activities, signal transduction pathways, or gene expression profiles, thereby affecting cell proliferation. The underlying mechanisms warrant further investigation.

### Effects of adding three different electrically charged amino acids on the synthesis quantity of α-casein

The raw materials for the synthesis of α-casein mainly originate from small-molecule oligopeptides and free amino acids in the blood. These raw materials enter the cells through selective absorption by the mammary epithelial cells, providing the material basis for the synthesis of α-casein. It can thus be seen that, as the precursors of milk proteins, the types and levels of free amino acids in the blood directly influence the synthesis quantity of α-casein in the mammary epithelial cells of dairy cows^33^. The experimental results demonstrate that an increase in amino acid concentration is associated with a gradual enhancement in α-casein synthesis. This suggests that the preferential or abundant availability of specific amino acids may enable targeted regulation of mammary cell function. Amino acids are actively and efficiently transported into the cells via specialized transporters, leading to significantly elevated amino acid metabolic flux and local concentrations within the glandular microenvironment—levels that surpass normal physiological conditions. This enrichment ensures adequate cellular uptake and facilitates the activation of downstream signaling and metabolic pathways, thereby optimizing the functional potential of these cells. Meanwhile, after the concentration difference of amino acids is generated inside and outside the cells, they are transmembrane transported into the cells by amino acid transporters, facilitating the increase in the synthesis of α-casein within the cells. The newly synthesized α-casein polypeptide chains within the cells are guided by signal peptides, chemically modified in the endoplasmic reticulum, and processed and packaged in the Golgi apparatus to form mature protein particles or vesicles, which fuse with the Golgi apparatus membrane and are secreted as vesicles. These secretory vesicles fuse with the cell membrane by exocytosis and release α-casein to the extracellular environment. This suggests that different amino acid patterns within the culture medium can influence the quantity of mammary epithelial cells and thereby regulate the synthesis amount of casein. Amino acids, as the fundamental units of protein synthesis, their electrical characteristics might also impact the elongation and folding of peptide chains, thereby affecting the rate and efficiency of protein synthesis.The function of proteins is to a large extent determined by their three-dimensional structure, and the electrical nature of amino acids also exerts a crucial influence on protein folding and stability. Polar amino acids are typically situated on the surface of proteins, interacting with aqueous solutions, which contributes to maintaining the solubility and stability of proteins and is involved in multiple physiological processes such as enzymatic activity and signal transduction in organisms; non-polar amino acids are usually located within proteins, forming a hydrophobic core, which helps sustain the three-dimensional structure and stability of proteins.

Chen Jiao^34^noted that acidic amino acids might influence the intracellular pH balance, thereby indirectly affecting protein synthesis. Acidic aspartic acid (Asp) and glutamic acid (Glu) might have an impact on the expression and activity of amino acid transporters, thereby influencing the uptake and utilization of amino acids. Wang Nan^35^proved that negatively charged amino acids might affect the formation of casein micelles by regulating the intracellular calcium ion concentration. Consistent with the significant enhancement of α-casein synthesis in MAC-T cells following glutamic acid supplementation observed in this experiment, glutamic acid is one of the predominant amino acids in milk proteins. Mepham^36^ demonstrated through studies measuring arteriovenous differences of free amino acids in plasma from lactating goat mammary glands that mammary uptake of glutamic acid closely matches its secretion into milk. This indicates a substantial contribution of glutamic acid to milk protein synthesis and suggests its potential to improve the quality of dairy products. Chen Jiao^34^concurrently substantiated that the non-polar and hydrophobic phenylalanine (Phe) can significantly influence the mRNA expression levels of casein synthesis genes CSN1S1, CSN2, and CSN3, as well as the synthesis of α-casein, which is in accordance with the outcome of this experiment where non-polar proline significantly augmented the synthesis quantity of α-casein. The α-casein molecule encompasses a considerable amount of non-polar amino acids, which play a crucial role in the structure of α-casein. They are linked with other amino acid residues via hydrophobic interactions, forming the stable structure of α-casein. Simultaneously, the hydrophobicity of non-polar amino acids also enables α-casein to interact with other hydrophobic substances, such as the oil-water interface, thereby conferring specific functional characteristics upon α-casein. In this experiment, polar uncharged serine exhibited a certain enhancing effect on intracellular casein synthesis. As one of the amino acid precursors necessary for the synthesis of α-casein, polar uncharged amino acids may impact the synthesis amount of α-casein through providing synthetic precursors, influencing cellular signal transduction, and promoting enzyme activity. Hence, the influence of the electrical property of amino acids on the synthesis of α-casein is multifaceted and may include its solubility, stability, folding, function, and protein engineering via electrochemical modification.

During the lactation period of dairy cows, particularly in high-yielding animals during peak lactation, the physiological demand for amino acids reaches its maximum. In addition to fulfilling the requirements of traditionally recognized limiting amino acids such as lysine and methionine, ensuring adequate dietary supply of proline, glutamic acid, and serine may enhance mammary cellular metabolic efficiency, thereby improving milk protein synthesis and increasing milk protein concentration.

### Effects of adding three different electrically charged amino acids on the synthesis gene of α-casein

Amino acids not only serve as substrate precursors for milk protein synthesis but also function as intracellular signaling molecules that regulate key biological processes, including protein translation and post-translational modifications such as phosphorylation. They activate specific intracellular amino acid-sensing pathways, thereby collectively contributing to the maintenance of systemic amino acid homeostasis, ensuring normal cellular function, enabling precise regulation of gene expression during protein synthesis, and participating in diverse physiological activities within the organism^37–39^. The addition of amino acids may influence the expression of amino acid transporters, thereby affecting the uptake and utilization efficiency of amino acids. This alteration could subsequently modulate the expression of CSN1S1 and CSN1S2 genes and impact α-casein synthesis. Furthermore, the electrical properties of amino acid side chains can affect protein structure and function^40^. Charged amino acids can interact with other molecules via ionic bonds, thereby influencing the stability and activity of proteins. Protein synthesis is a complex process involving gene transcription and translation. The regulation of gene expression encompasses multiple aspects such as DNA sequences, transcription factors, and epigenetic modifications. The electrical nature of amino acids may indirectly affect the regulation of gene expression by influencing the three-dimensional structure of proteins and subsequently their functions, and further by influencing protein interactions and functions to indirectly impact these regulatory mechanisms^41^. Different electrically charged amino acids may affect the transcription level of the α-casein gene CSN1S1 by regulating the expression of transcription factors^42^. The experimental results indicate that the three amino acids with distinct electrical properties significantly elevated the expression levels of the α-casein synthesis-related genes CSN1S1 and CSN1S2. The promoting effect of polar uncharged serine on gene expression is remarkable, being capable of effectively enhancing the expression levels of the CSN1S1 and CSN1S2 genes, non-polar proline has the most significant effect.

As a precursor substance for protein synthesis, serine plays a crucial role in cell metabolism and signal transduction, thereby facilitating the synthesis of casein. Pszczolkowski^43^et al. investigated the synergistic action of insulin and essential amino acids and discovered that serine has a stimulatory effect on the mTORC1 signaling pathway, thereby augmenting protein synthesis. The multiple functions of serine in metabolic pathways (such as its involvement in one-carbon metabolism) may also exert an indirect influence on the synthesis of milk proteins. Although polar uncharged glutamine is also implicated in the regulation of the mTOR pathway, its mode of action might be as a substrate for indirect catalysis or it might require the synergy of multiple amino acids to achieve the optimal outcome^44^. Furthermore, glutamine is not merely a substrate for protein synthesis but also indirectly influences the activity of mTOR through modulating cellular redox status, energy metabolism, and autophagy, among other pathways^45^. In mammary epithelial cells, both non-polar isoleucine and leucine can influence the rate of milk protein synthesis by regulating the phosphorylation of factors such as mTOR and 4EBP1 in the mTOR signaling pathway, and concurrently reduce the abundance of proteasome proteins, ubiquitinated proteins and the rate of protein degradation^46^. Some scholars^47,48^have demonstrated that when essential amino acids such as leucine are individually added to the culture medium, the gene expression levels of mTOR, S6K1 and four types of caseins (CSN1S1, CSN1S2, CSN2 and CSN3) in bovine mammary epithelial cells (BMECs) are significantly upregulated. Qi H^49^et al. has pointed out that leucine ultimately promotes the expression of genes related to casein synthesis by activating the phosphorylation of signal proteins in the mTOR signaling pathway. Likewise, Chen Jiao^34^has also demonstrated that non-polar phenylalanine (Phe) can enhance the activity of the mTOR signaling pathway by regulating the phosphorylation levels of 4EBP1 and eIF2α, thereby facilitating the synthesis of α-casein in bovine mammary epithelial cells. Therefore, non-polar amino acids can serve as signaling molecules to participate in intracellular signal transduction processes and influence the regulatory mechanisms of gene expression. This is in line with the significant effect of the sole addition of non-polar proline on casein synthesis genes in this experiment.

Negatively charged glutamic acid significantly enhances the expression of the α-casein gene. As a fundamental precursor in protein synthesis, glutamic acid regulates genes associated with casein production. According to research by Brosnan^50^ et al., glutamic acid primarily functions as a metabolic intermediate in mammary epithelial cells, playing a critical role in cellular metabolism and modulating the expression of genes involved in milk protein synthesis. Therefore, glutamic acid may indirectly influence casein synthesis by regulating gene expression mechanisms. The side chains of negatively charged amino acids bear a negative charge at physiological pH and are situated close to the transcription factor binding sites in the gene promoter region. They might affect the binding of transcription factors via electrostatic interactions. The negative charge could potentially attract positively charged transcription factors, enhancing the binding affinity of transcription factors and thereby facilitating the transcription of CSN1S1 and CSN1S2 genes. During the process of cellular signal transduction, charged amino acids may participate in the modification and transmission of signal molecules. Negatively charged amino acids may undergo phosphorylation modification, thereby altering the activity and functionality of signal molecules and subsequently influencing the expression regulation of CSN1S1 and CSN1S2 genes^40^.

## Materials and methods

### Test main instruments and reagents

Constant-temperature water bath (domestic), high-pressure steam sterilizer (Shanghai Bosun Medical), fully automatic microplate reader (Thermo Fisher Scientific, USA), high-speed refrigerated centrifuge (Sigma, Germany), ultraviolet spectrophotometer (Beckman), Quantstudio multiplex real-time fluorescence quantitative PCR instrument (Life Technologies, USA), incubator, constant-temperature CO2 incubator (Haier Bio-Medical), super-clean bench (Shanghai Bosun Medical), Countess 3 FL fully automatic cell counter (Thermo Fisher Scientific, USA).

DMEM high glucose medium (C11965500BT), fetal bovine serum (Unheat-inactivated, 10099-141), and trypsin (25200-056) were procured from Gibco; double antibiotics (SV30010) and phosphate buffered saline (SH30256.01) were obtained from Hyclone; L-glutamic acid (G8415-100G), L-proline (P5607-25G), L-serine (S4311-25G), and dimethyl sulfoxide (D4540-100mL) were acquired from Sigma; Cell Counting Kit-8 (C0037) was purchased from Beyotime Biotechnology Co., Ltd.; the enzyme-linked immunosorbent assay (ELISA) kit (JL22469) was obtained from Jianglai Biotechnology; TRIzol^®^ Plus RNA Purification Kit (Invitrogen item number: 12183-555), RNase-Free DNase Set (Qiagen item number: 79254), and Power SYBR^®^ Green PCR Master Mix (Roche item number: 4913914001).

### MAC-T cells

The bovine mammary epithelial cells line (MAC-T cells) was procured from the cell bank of Qingqi (Shanghai) Biotechnology Development Co., Ltd. After continuous subculture, the cells exhibited stable adherent growth characteristics, presenting as a monolayer with a cobblestone-like mosaic arrangement. The cells were closely connected with clear boundaries. The proliferation activity was stable and the cells could be stably cultured for more than 30 generations. The cell population maintained over 95% morphological homogeneity, and no abnormal vacuolization or fibrosis was observed. The cells had typical morphological characteristics of bovine mammary epithelial cells and were suitable for subsequent experiments.

### Test methods

The experiment employed a multi-factor and multi-level random experimental design to establish three amino acid pattern groups, each with seven levels. MAC-T cells were cultured in vitro using a medium containing 10% fetal bovine serum (10% FBS DMEM) until they adhered to the wall. After that, the cells were subjected to starvation culture in serum-free medium for 12 h. The cells in the serum-free medium group were regarded as the blank control group (CK group, 0× group), and the medium supplemented with amino acids of three electrical properties and different levels (Table 1) was used as the experimental group. Each group had three replicates.

**Table 1:** Levels of amino acids added to the test medium (mmol/L).

Items	Groups						
	0×	0.25×	0.5×	1×	2×	4×	8×
Proline	0.00	0.52	1.04	2.09	4.17	8.35	16.69
Glutamic acid	0.00	0.77	1.53	3.06	6.13	12.25	24.50
Serine	0.00	0.50	0.99	1.97	3.93	7.86	15.71

#### Mammary epithelial cell culture in dairy cows

MAC-T cells were placed in DMEM high-glucose growth medium supplemented with 10% fetal bovine serum and cultivated in an incubator at 37 °C with 5% CO_2_. The medium was changed periodically to monitor the cell state. Every 2 to 3 days, the cells were subcultured at a ratio of 1:6 with a seeding density of 0.75 × 10^6^ cells per T25 flask.

#### Cell proliferation rate assay

The proliferation rate was determined using the CCK8 method. At various time points (4 h, 6 h, 12 h, 24 h, and 48 h), the proliferation rate of the cells was detected via the CCK8 kit. The absorbance value was measured at a wavelength of 450 nm by an enzyme-labeled immunosorbent assay reader. The cell proliferation rate was calculated by the following formula:

Cell proliferation rate (%) = (OD450nm value of the experimental group - OD450nm value of the blank control group) / (OD450nm value of the control group - OD450nm value of the blank control group) × 100%.

#### Measurement of α-casein synthesis

After 12 h of amino acid supplementation, the culture medium was collected, centrifuged at 1000 r/min for 10 min, and the supernatant and cells were gathered. The cell pellet was disrupted using an ultrasonic cell disruptor. All the collected samples were determined for the levels of α-casein in the supernatant and intracellularly in accordance with the ELISA kit instructions. The specific procedures are as follows:

1. MAC-T cells were inoculated in T25 flasks at a density of 0.7 × 10^4^ cells per flask, and 6 mL of DMEM medium containing 10% FBS was added to each flask. The flasks were then placed in a 37 °C, 5% CO_2_ incubator for 8–10 h to enable the cells to adhere.
2. Discard the old medium in the culture flasks, and add 6 mL of serum-free medium to each flask. Continue the culture for 12 h to eliminate the interference of unknown factors in the serum on the experiment.
3. Discard the old medium in the culture flasks. According to the experimental design, add 6 mL of serum-free medium containing different levels of Glu (0.77 mmol/L, 1.53 mmol/L, 3.06 mmol/L, 6.13 mmol/L, 12.25 mmol/L, 24.50 mmol/L), Pro (0.52 mmol/L, 1.04 mmol/L, 2.09 mmol/L, 4.17 mmol/L, 8.35 mmol/L, 16.69 mmol/L), and Ser (0.50 mmol/L, 0.99 mmol/L, 1.97 mmol/L, 3.93 mmol/L, 7.86 mmol/L, 15.71 mmol/L) to each flask respectively. Each treatment group has three replicates. The group with 6 mL of serum-free medium added is set as the blank control group (CK group).
4. Place the T25 flask in a CO_2_ incubator at 37 °C with 5% CO_2_ and incubate for 12 h. Subsequently, collect the cell culture supernatant and cell samples, and centrifuge both at 1000 r/min for 10 min. For the cell pellet, resuspend it in 2 mL of pre-chilled PBS and then disrupt the cell samples using an ultrasonic cell disruptor.
5. Set up standard wells, blank wells, and sample wells (cell culture supernatant samples and cell disruption samples). Add 50 µL of standards of different concentrations to each standard well. Add 50 µL of the cell supernatant and disrupted cell samples to be tested to the sample wells.
6. With the exception of the blank wells, add 100 µL of horseradish peroxidase (HRP)-labeled detection antibody to each well of the standard and sample wells. Seal the reaction wells with a sealing membrane and incubate in a 37 °C water bath in the dark for 60 min.
7. Discard the liquid, blot dry on absorbent paper, fill each well with washing solution (350 µL), stand for 1 min, flick off the washing solution, blot dry on absorbent paper, and repeat this washing process 5 times. Add 50 µL of substrate A and 50 µL of substrate B to each well, and incubate in the dark at 37 °C for 15 min.
8. Add 50 µL of the stop solution to each well. Within 15 min, measure the OD values of each well at a wavelength of 450 nm.
9. Employ an enzyme-labeled immunosorbent assay (ELISA) reader to determine the absorbance (OD) at a wavelength of 450 nm and calculate the sample concentration. Plot a standard curve with the concentration of the standard substances as the ordinate and the OD values as the abscissa. Calculate the concentration of α-casein in the sample based on its OD value using the standard curve.

#### Measurement of proline, glutamic acid, and Serine on the expression of genes related to α-casein synthesis

Upon the completion of the culture, cells were collected in 1.5 mL EP tubes, and 400 µL of RNA lysis buffer was added. Total RNA was extracted, and its concentration as well as the OD260nm/OD280nm ratio were determined. cDNA was synthesized through reverse transcription. Quantitative PCR primers were designed using Primer Premier 6.0 and Beacon Designer 7.8 software and subsequently synthesized. The primer sequences and parameters are presented in Table 2. Each sample was replicated three times, and the relative expression levels of each gene were statistically analyzed by 2^(Ct ^reference gene^ - Ct ^target gene^).

**Table 2:** Primer sequences of genes related to α-casein synthesis.

Genes	Gen Bank	Primer Sequences	
CSNIS1	NM_181029.2	Forward	TCAACCCAGCTTGCTGCTTCTTCC
		Reverse	GCCTAGCAAGAGCAACAGCCACAA
CSN1S2	NM_174528.2	Forward	AGCAGCTCTCCACCAGTGAGGAAA
		Reverse	TGGGGCAAGGCGAATTTCTGGT
β-actin	NM_173979.3	Forward	TCCATCGTCCACCGCAAATGCT
		Reverse	TGCTGTCACCTTCACCGTTCCA

### Statistical analysis

The original data of the experiment were processed for calculation and analysis using Excel 2017 software. The fluorescence quantitative data were statistically analyzed by the 2-ΔΔCt method to assess the relative expression level of the target gene mRNA in MAC-T cells. The post-processed experimental data were subjected to one-way ANOVA using the ANOVA process of SAS9.0 software, and multiple comparisons were carried out using Duncan’s method, with *P* < 0.01 considered as extremely significant differences. The bar charts of cell proliferation rate, α-casein content, and target gene expression level were plotted using Origin7.5.

## Conclusions

This experiment systematically investigated the influences of three amino acids with different electrical properties (Pro, Glu, and Ser) on the proliferation rate of MAC-T cells, the synthesis of α-casein, and gene expression through a series of experiments. The results demonstrated that the electrical property and concentration of amino acids, as well as different time points, played a crucial role in regulating the activity of mammary cells and the synthesis of milk proteins. When 16.69 mmol/L Pro, 24.50 mmol/L Glu, and 0.99 mmol/L Ser were added respectively at a culture time of 12 h, they could not only significantly enhance cell proliferation but also significantly up-regulate the expression levels of MAC-T cells α-casein synthesis-related genes. Furthermore, the significance of the three amino acids with different electrical properties in affecting the synthesis level of α-casein was in the order of Pro > Glu > Ser. This experiment establishes a novel theoretical foundation for formulating more precise amino acid-based nutritional strategies in dairy cows, offering a promising avenue to enhance their production performance through targeted dietary regulation of specific amino acids. Future studies will aim to validate these findings in animal models and further investigate the underlying signal transduction mechanisms involved.

## Supplementary Information

Below is the link to the electronic supplementary material.

Supplementary Material 1

Supplementary Material 2

Supplementary Material 3

## Data Availability

All data generated or analysed during this study are included in this published article and its supplementary information files.

## Abbreviations

MAC-T cells: Bovine mammary epithelial cells line
RT-qPCR: Real-time quantitative PCR
FBS: Fetal bovine serum
ELISA: Enzyme-linked immunosorbent assay
mTOR: Mammalian target of rapamycin
Pro: Ploline
Glu: Glutamic acid
Ser: Serine
